# Late-Stage Löffler's Endocarditis Mimicking Cardiac Tumor: A Case Report

**DOI:** 10.3389/fcvm.2020.589212

**Published:** 2020-10-29

**Authors:** Takafumi Koyama, Hiroyuki Yamamoto, Manabu Matsumoto, Jun Isogai, Tadashi Isomura, Shinji Tanaka

**Affiliations:** ^1^Department of Cardiology, Shonan Fujisawa Tokushukai Hospital, Kanagawa, Japan; ^2^Department of Cardiovascular Medicine, Narita-Tomisato Tokushukai Hospital, Chiba, Japan; ^3^Department of Pathology, National Cerebral and Cardiovascular Center, Suita, Japan; ^4^Department of Radiology, Asahi General Hospital, Asahi, Japan; ^5^Department of Cardiovascular Surgery, IMS Katsushika Heart Center, Tokyo, Japan

**Keywords:** hypereosinophilic syndrome, Löffler's endocarditis, cardiac thrombus, cardiac magnetic resonance, endomyocardial fibrosis

## Abstract

Löffler's endocarditis (cardiac involvement in hypereosinophilic syndrome) is rare yet life-threatening if left untreated. We describe a case of hypereosinophilic syndrome presenting as a cardiac mass with an abnormal electrocardiogram. Diagnostic studies of the cardiac mass strongly suggested a malignant cardiac tumor invading the papillary muscle. Thus, excision of the cardiac mass and endomyocardial resection with mitral valve replacement were successfully performed. Pathology revealed various stages of thrombosis and irreversible myocardial damage caused by eosinophilic infiltration with no malignancy, leading to the correct diagnosis of late-stage Löffler's endocarditis. The subsequent combination of anticoagulation and corticosteroids was effective with a favorable outcome. This case highlights pitfalls in multimodality imaging of cardiac thrombus and the clinical significance of considering Löffler's endocarditis in the diagnostic work-up of a cardiac mass.

## Introduction

Hypereosinophilic syndrome (HES) is an uncommon group of diseases characterized by persistent hypereosinophilia with eosinophil-mediated multiple organ involvement (1). Diagnosis of HES is based upon the following criteria; an absolute eosinophil count >1,500 cells/μL in the peripheral blood for at least 1 month, organ damage and/or dysfunction caused by eosinophilic infiltration, and exclusion of potential causes of secondary eosinophilia including allergic diseases and parasitic infections.

Löffler's endocarditis (LE; cardiac involvement in HES) progresses through three stages, namely acute necrotic stage, thrombotic stage, and fibrotic stage, which may overlap (2, 3). LE exhibits various cardiac findings depending on the severity and extent of the endo-/myocardial damage caused by eosinophilic infiltration (4). During the acute necrotic phase, patients present with symptoms and signs of left-sided heart failure (HF). During the thrombotic phase, patients develop cardiac thrombi and present with symptoms and signs based on embolic events such as stroke, or mass effects including secondary mitral regurgitation (MR) and ventricular cavity obliteration, respectively. During the fibrotic phase, patients develop restrictive cardiomyopathy and have symptoms and signs of right-sided HF. Uncommon cases present with symptoms and signs of coronary arteritis, aortic valve stenosis, and pericarditis (5, 6).

If left untreated, serious complications such as valvular regurgitation or restrictive cardiomyopathy may occur at an advanced stage. However, an early correct diagnosis is particularly difficult because of the subclinical presentation and the lack of characteristic signs in early disease stages.

## Case Presentation

A 77-year-old man was referred to our hospital for the evaluation of suspected cardiac abnormalities, which were detected on electrocardiogram (ECG) during a medical checkup.

The patient had a 3-year history of hypereosinophilia, for which etiologies remained unknown despite thorough diagnostic evaluation.

**Figure 1:**
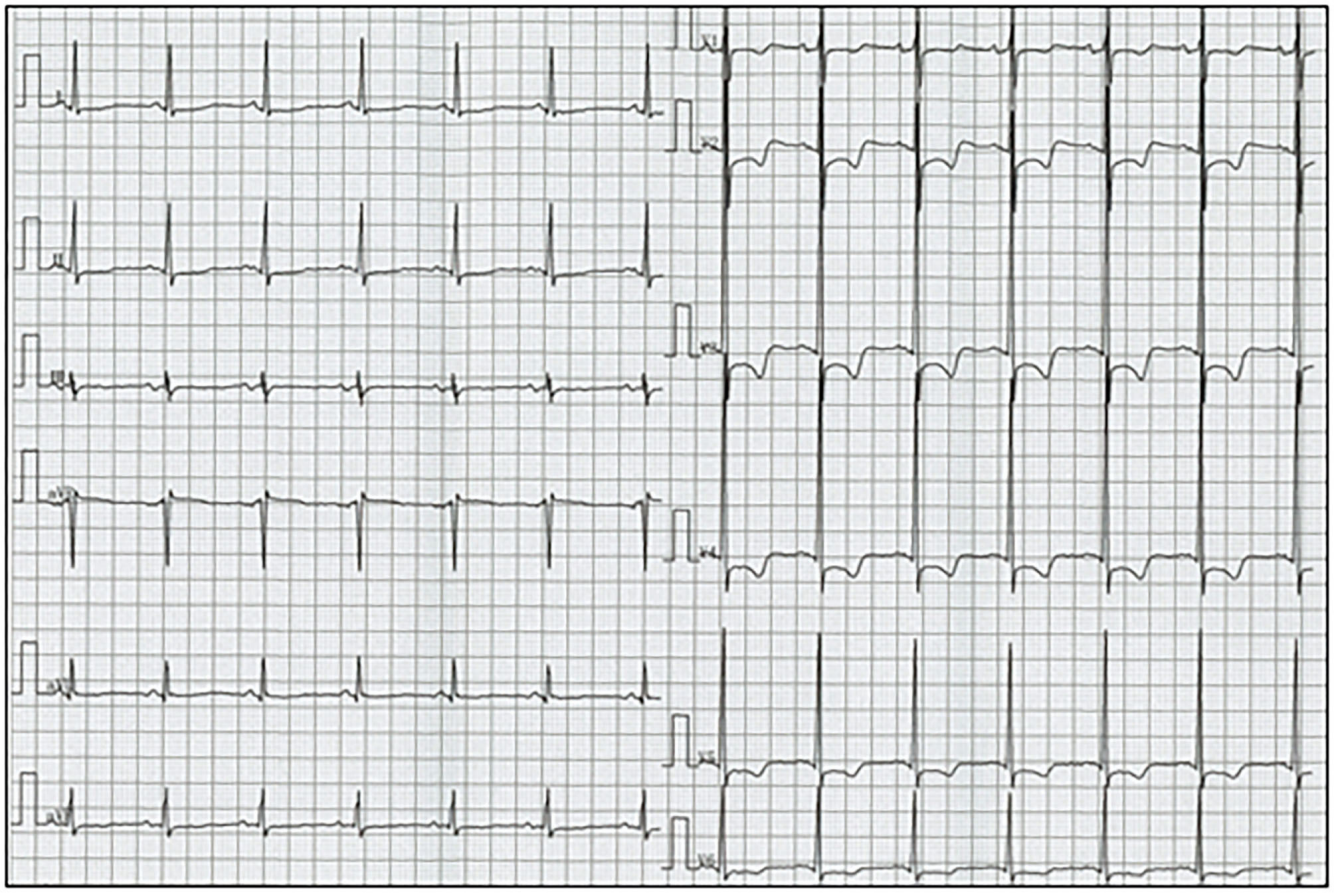
Electrocardiogram reveals sinus rhythm and ST segment depression with T-wave inversion in leads V1-6.

**Figure 2:**
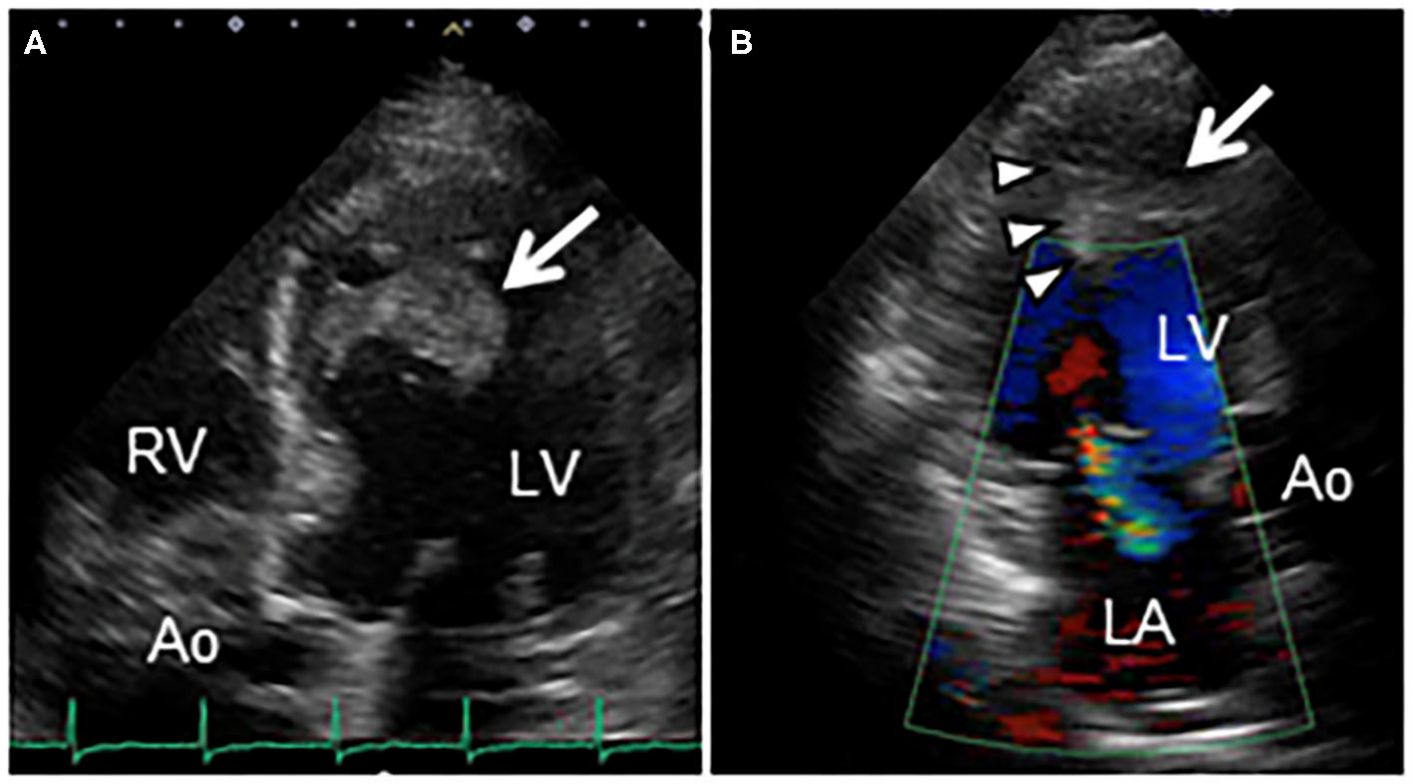
**(A)** Transthoracic echocardiography in apical 5-chamber view reveals an echo-dense mass (30 × 39 mm, arrow) attached to the mid-anteroseptal wall of the left ventricle, extending to the anterolateral papillary muscle. **(B)** Color Doppler echocardiography in apical 3-chamber view reveals a cardiac mass (arrow), and entrapment of the anterolateral papillary muscle (arrowheads), causing moderate mitral regurgitation. Ao, Aorta; LA, Left atrium; LV, Left ventricle; RV, Right ventricle.

**Figure 3:**
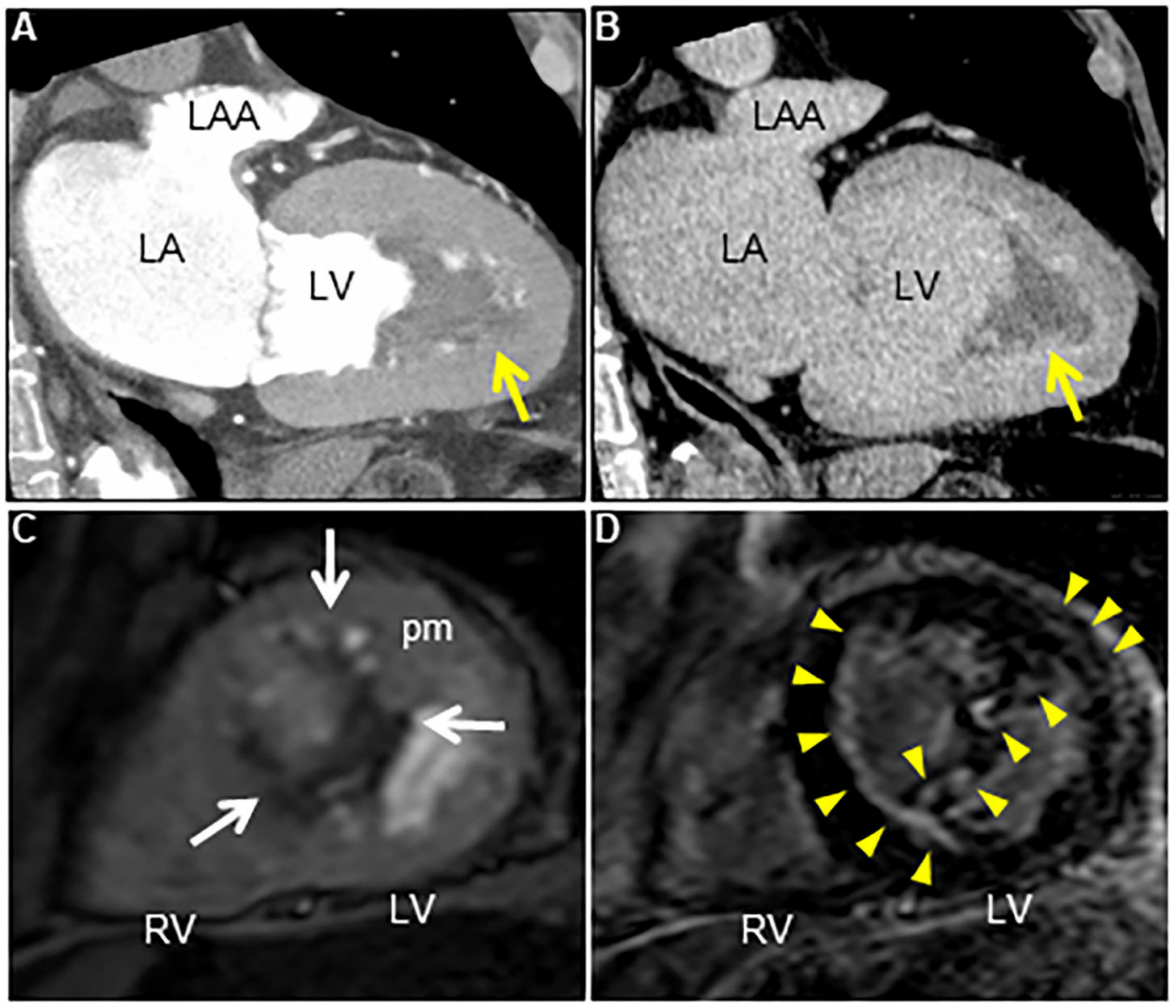
**(A)** Contrast-enhanced computed tomography scan at early phase reveals an irregular cardiac mass attached to the left ventricle in several locations (arrow). **(B)** Note the reduced mass with a delayed contrast enhancement at late phase (arrow). **(C)** Axial dynamic cardiac magnetic resonance imaging reveals a low echogenic left ventricular mass attached to the anteroseptal and inferior walls, and the anterolateral papillary muscle (arrows). **(D)** Late gadolinium enhancement image reveals subendocardial enhancement in anteroseptal and inferior walls, mid-wall enhancement in lateral wall, and heterogeneous enhancement within the cardiac mass and the adjacent anterolateral papillary muscle (arrowheads). LA, Left atrium; LAA, Left atrial appendage; LV, Left ventricle; pm, Papillary muscle; RV, Right ventricle.

His vital signs were stable. He had no symptoms or signs of HF. Laboratory test results revealed elevated white blood cells (19,400/μL) with eosinophilia (absolute eosinophilic count: 9,120/μL; 47% in differential), and brain natriuretic peptide (BNP) levels (187.6 pg/mL, normal range: 0–18.4 pg/mL). Both C-reactive protein (CRP) and cardiac troponin I (cTnI) levels were within the normal range. ECG revealed ST segment depression with T-wave inversion in the precordial leads (Figure 1), which were not observed 3 years ago. Echocardiography revealed normal chamber size with normal left ventricular ejection function of 69%, despite grade II diastolic dysfunction. A large, heterogeneous cardiac mass (30 × 39 mm, arrow) with a stalk attached to the septum was noted in the apex of the left ventricle (Figure 2A, Supplementary Video 1). Color Doppler echocardiography revealed that the mass had reached the anterolateral papillary muscle (ALPM), resulting in moderate MR (Figure 2B, Supplementary Video 2). Coronary angiography was unremarkable. Contrast-enhanced computed tomography revealed a large, low attenuation intracardiac mass with several attachments to the apex of the left ventricle. Note an inhomogeneous and peripheral delayed enhancement as if the mass size had reduced (Figures 3A,B). Cardiac magnetic resonance (CMR) imaging further confirmed the cardiac mass, with myocardial tissue properties (Figures 3C,D). A short-axis image of the mid-ventricle revealed considerable late gadolinium enhancement (LGE) found subendocardially in the anteroseptal and inferior wall, and in the midportion of the lateral wall. Moreover, heterogeneous enhancement was also seen within the mass and the adjacent ALPM, strongly suggesting a cardiac tumor with surrounding invasion. Differential diagnoses included primary neoplasm (angiosarcoma or malignant lymphoma), secondary neoplasm (metastatic breast, lung or renal carcinoma), benign tumor (myxoma or hamartoma), vegetation, and thrombus.

**Figure 4:**
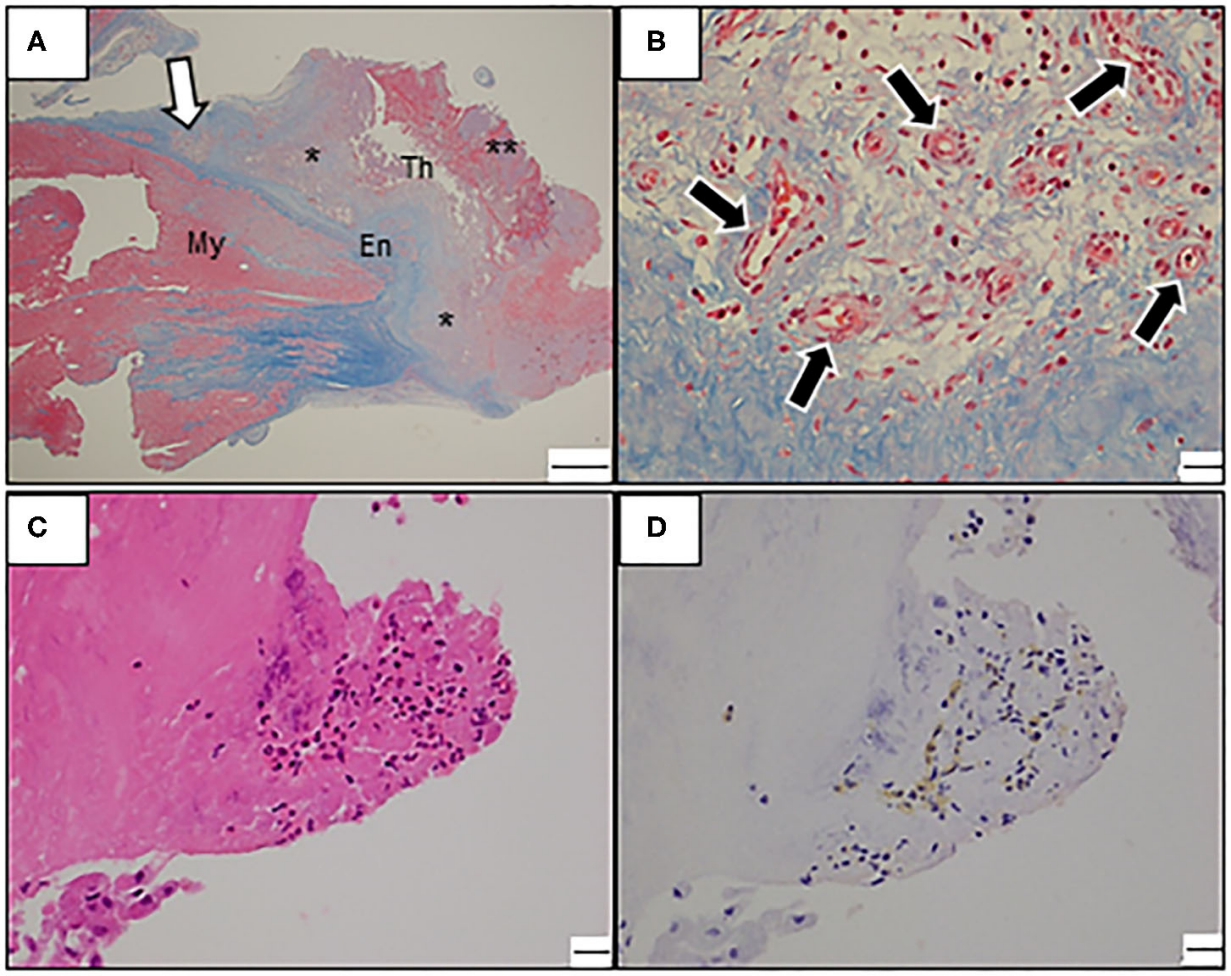
**(A–D)** Photomicrograph of the resected mass. **(A)** Low-power view with Masson trichrome stain reveals endocardial fibrosis and intracardiac thrombosis at various stages. Note the mixed thrombus consisting of well-organized thrombus (asterisk) and fresh thrombus (double asterisk). En, Endocardium; My, Myocardium; Th, Thrombus; Bars: 1 mm. **(B)** A high-power view reveals proliferating neovascularization (black arrows) with inflammatory infiltrates in the border zone of endocardial fibrosis and mural thrombus (magnified view around white arrow in **A**). Bars: 20 μm. **(C)** A high-power view with hematoxylin-eosin stain reveals focal eosinophilic infiltrate in the endocardium. Bars: 20 μm. **(D)** Immunostaining with an antibody against the major basic protein. Bars: 20 μm.

Owing to the increased risk of exacerbation of MR or systemic embolization, the patient underwent surgical resection of the cardiac mass. In the intraoperative view, the 4 cm-sized yellowish-white mass was integrated with the left ventricular endocardium. Moreover, the boundaries between the mass and the endocardium as well as ALPM were poorly demarcated and rigid. Thus, endomyocardial resection and mitral valve replacement (MVR) with bioprosthetic valve (Magna Ease, 25 mm), combined with resection of the affected ALPM were performed in addition. Pathological examination of the resected mass revealed mixed thrombus consisting of fresh, organizing, or organized thrombus, with no malignancy (Figures 4A,B). Moreover, the resected endomyocardium and the ALPM demonstrated various changes from granulation to severe fibrosis, suggesting a fibrotic stage. Although eosinophil infiltrations were observed in the endocardium, immunostaining for major basic protein, which is one of eosinophil-specific cardiotoxic granules, showed no remarkable extracellular deposition, indicating no evidence of eosinophilic activation (Figures 4C,D). These findings led to a correct diagnosis of late-stage LE.

**Figure 5:**
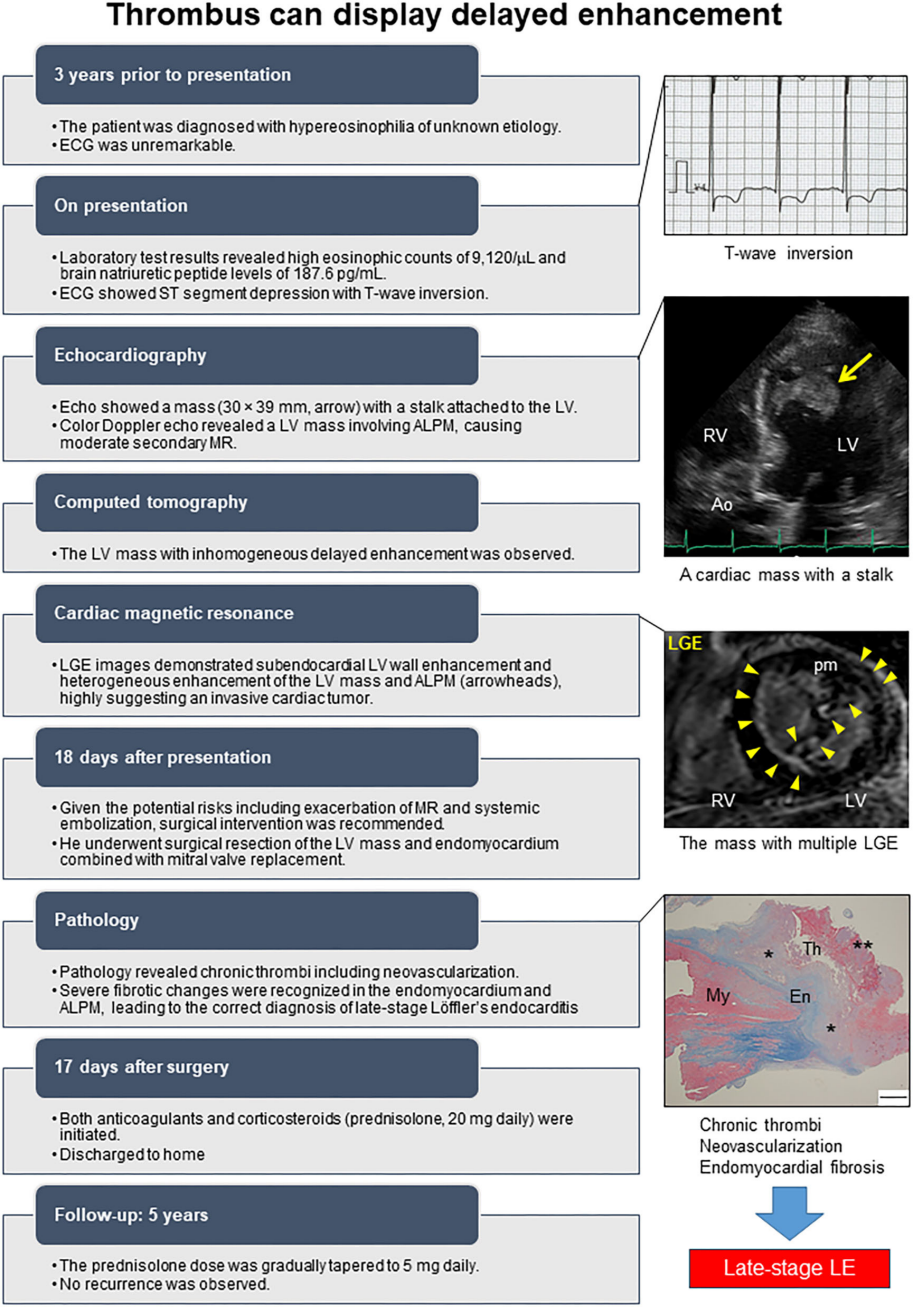
Timeline of the case. ALPM, Anterolateral papillary muscle; Ao, Aorta; Echo, Echocardiography; ECG, Electrocardiogram; En, Endocardium; LGE, Late gadolinium enhancement; LV, Left ventricle; MR, Mitral regurgitation; My, Myocardium; pm, Papillary muscle; RV, Right ventricle; Th, Thrombus. The asterisk denotes well-organized thrombus, and double asterisk denotes fresh thrombus.

The postoperative course was uneventful. Subsequently an anticoagulant and a high dose of corticosteroid (prednisolone 20 mg daily) were initiated. The prednisolone dose was gradually tapered to 5 mg daily. No recurrence was observed during 3-year follow-up. As a supplement, we present a summarized illustration of the case presentation (Figure 5).

## Discussion

Herein, we describe a case of histologically proven late-stage LE masquerading as a cardiac tumor. This case may provide several precious clinical lessons.

First, our case exhibited an atypical LE characterized by a cardiac mass, which was initially mistaken as an invasive cardiac tumor. Although LE and cardiac tumor are similar in terms of cardiac mass development, they have different prerequisite treatment strategies. Thus, the LE-associated cardiac thrombus must be clinically differentiated from a cardiac tumor.

Although laboratory tests are not specific to LE, they can sometimes be useful for detecting myocarditis or as indicators of its activity; CRP can be considered a well-known inflammation marker. Cardiac enzyme is a specific indicator of myocardial necrosis, and BNP is another marker of cardiac damage owing to HF or restrictive physiology (7). In our case, normal CRP and cTnI levels and elevated BNP levels were consistent with late-stage LE.

Echocardiography is first-line imaging modality for detection of endocardial damage of HES. During the acute inflammatory phase, echocardiography shows few significant findings. However, ventricular wall thickening corresponding to myocardial edema can be observed if the inflammatory infiltration is severe. The characteristic echocardiographic findings in LE include apical cavity obliteration of the ventricles caused by echogenic material with preserved ventricular ejection fraction (8). Its echocardiographic findings are very similar to those of apical hypertrophic cardiomyopathy or left ventricular non-compaction, leading to misdiagnosis. In addition, severe MR owing to limited posterior mitral leaflet motion, biatrial dilatation despite normal ventricular chamber size, or restrictive filling pattern can be recognized (9). In our case, echocardiography demonstrated a large heterogeneous mass with a stalk attached to the ventricular septum. Such atypical morphological characteristics identified on echocardiography might lead to misdiagnosis. Recently, three-dimensional speckle tracking echocardiography allows identifying abnormal findings of both atrial structural and functional remodeling even in asymptomatic LE patients during the acute necrotic phase, which can be valuable for an early diagnosis of LE (10, 11).

CMR is another essential non-invasive imaging tool for evaluating tissue properties and detecting thrombus in LE, and it is becoming the gold standard. Compared with transthoracic echocardiography (23% ± 12% and 96% ± 4%, respectively) and transesophageal echocardiography (40% ± 14% and 96% ± 4%, respectively), contrast-enhanced CMR has excellent sensitivity and specificity for detecting left ventricular mural thrombi (88% ± 9% and 99% ± 2%, respectively) (12). CMR allows the visualization of myocardial inflammation and fibrosis and the differentiation of inflammation from fibrosis based on the enhancement on LGE imaging (2). Typical CMR findings for LE include the following: CMR using the steady-state free precession technique shows diffuse subendocardial thickening of the ventricle. T2-weighted imaging shows diffuse subendocardial hyperintensity signal in the ventricle, suggesting the presence of inflammatory edema. The LGE imaging study demonstrates a characteristic three-layer appearance in the biventricular apex composed of the myocardium (outermost layer), diffuse subendocardial delayed enhancement indicating either wall edema or fibrosis or both (middle layer), and overlying hypointense area, consistent with mural thrombus (innermost layer) (13, 14). Moreover, several recent novel quantitative CMR imaging techniques, such as feature tracking-based strain analysis and T2-mapping, allow myocardial deformation analysis and myocardial edema assessment, respectively (15). Thus, multiparametric CMR is very useful for comprehensive tissue characterization, including assessment of myocardial perfusion, edema, and fibrosis, and can allow non-histological diagnosis, staging, and treatment monitoring of LE (16).

However, in our case, cardiac thrombus showed patchy distribution, not a null signal on LGE imaging, which is considered to be its more conventional finding; to the best of our knowledge, this is the first such report of a case with LE. The absence of LGE in a cardiac mass is believed to be one of the key findings in distinguishing thrombus from a tumor, since it implies avascular structures (17). However, a thrombus can have various signal intensities on CMR imaging since thrombus composition depends on its origin and age. While fresh thrombus includes a different fibrin-platelet rich composition, chronic thrombus can include neovascularization, calcification, and fibrotic or connective tissue deposition. There is some strong evidence to suggest that organized thrombosis may display heterogeneous enhancement on LGE imaging (18), supporting this notion. Also, peripheral delayed enhancement in contrast-enhanced computed tomography might have reflected a similar phenomenon.

However, the LV mass was notable despite the absence of LV wall motion abnormalities in our case; based on the presence of hypereosinophilia, the findings suggested endocardial damage owing to eosinophilic infiltration. These mismatched findings might be crucial for correctly diagnosing a rare disease. Endomyocardial biopsy remains the gold standard to diagnose LE and might provide more information for the preoperative diagnosis of LE in our case.

Second, combined surgical interventions and sequential medical treatments were feasible with favorable outcomes in our case. The treatment of LE consists of three strategies: suppression of tissue eosinophilic infiltration, counteracting inflammation, and prevention of fibrosis and thrombus formation. In addition, specific treatment is required according to the underlying cause of HES and the disease stage in each case. Based on previous case reports and a small number of case series (2), therapeutic interventions are initiated in a stepwise manner, usually starting with supportive care for HF, followed by medical treatment, and finally surgical intervention. However, the treatment outcome depends on the disease stage. Conventionally, a combination of immunosuppressive treatments including corticosteroids and anticoagulants is very effective in the control of early-stage LE (19). Similarly, surgical intervention can be avoided in many cases of LE with valvular involvement (often with mitral valve, sometimes aortic valve) (5, 20).

If the clinical picture is progressive or the disease is unresponsive to medical treatment, the disease is highly likely to be in an irreversible stage (21). However, no clear guidelines exist for treatment of late-stage LE, which is similar to the endomyocardial fibrosis (EMF). Ultimately, it is believed that orthotopic heart transplantation can be the only curative treatment for EMF. Although inconclusive owing to limited experience, endocardial decortication with/without atrioventricular valve repair or replacement is an acceptable option, which can improve symptoms and long-term survival in patients with EMF (22).

In the current case, endomyocardial resection with MVR, and subsequent combination of anticoagulation and corticosteroids were also effective. In fact, our patient could not receive proper treatment as described above. Retrospectively, considering that our case was in the histologically proven irreversible stage complicated by secondary MR, which indicated drug resistance, the surgical intervention might have made sense. However, there are great concerns about the increased risk of obstructive valve thrombosis, the long-term durability of biological valves, and the recurrence of fibrosis (23). The efficacy of anticoagulants and corticosteroids remains controversial. Further investigation is needed.

Finally, ECG monitoring was a helpful screening test for detecting LE.

Although most patients present with symptoms and signs of HF, our patient exhibited an asymptomatic cardiac mass with ECG abnormalities. A review of 65 cases demonstrated T-wave inversion on the ECG in 35% of cases, which was strongly associated with cardiac dysfunction (24). ECG monitoring is also effective for suspecting LE in our case.

The leading cause of morbidity and mortality in patients with HES is cardiac dysfunction. However, the development of cardiac involvement is unpredictable because there is not always a positive association between circulating eosinophil levels and eosinophil-mediated cardiac damage. Moreover, unawareness of this rare clinical entity, and non-specific symptoms is frequently related to missed or delayed diagnosis, leading to disease progression. Given the limited treatment approach in an irreversible stage, making an earlier diagnosis is crucial for appropriate treatment of LE.

**Figure 6:**
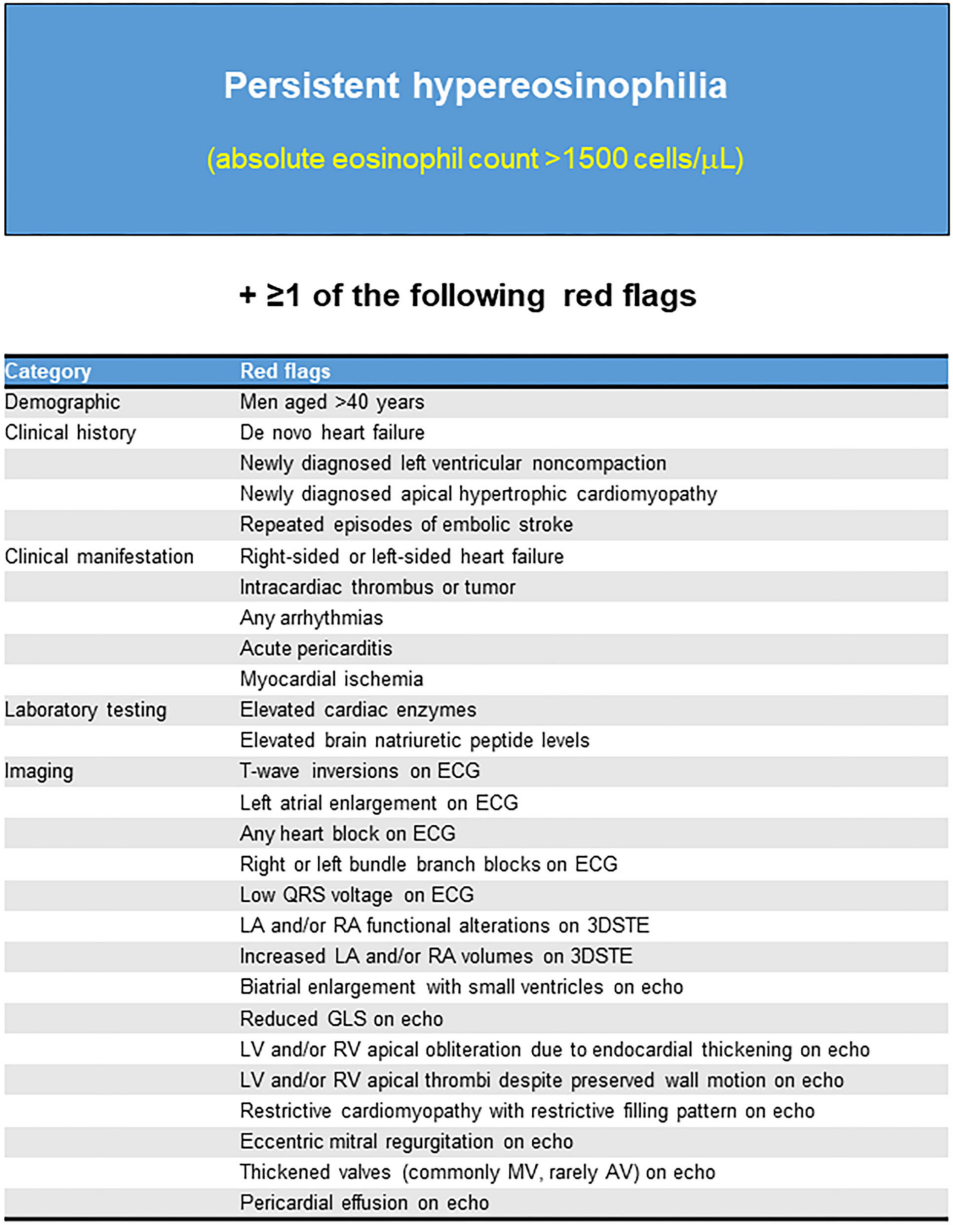
Red flags that should be highly suspected of Löffler's endocarditis. ECG, Electrocardiogram; Echo, Echocardiography; 3DSTE, Three-dimensional speckle tracking echocardiography; LA, Left atrium; RA, Right atrium; GLS, Global longitudinal strain; LV, Left ventricle; RV, Right ventricle; MV, mitral valve; AV, aortic valve.

**Figure 7:**
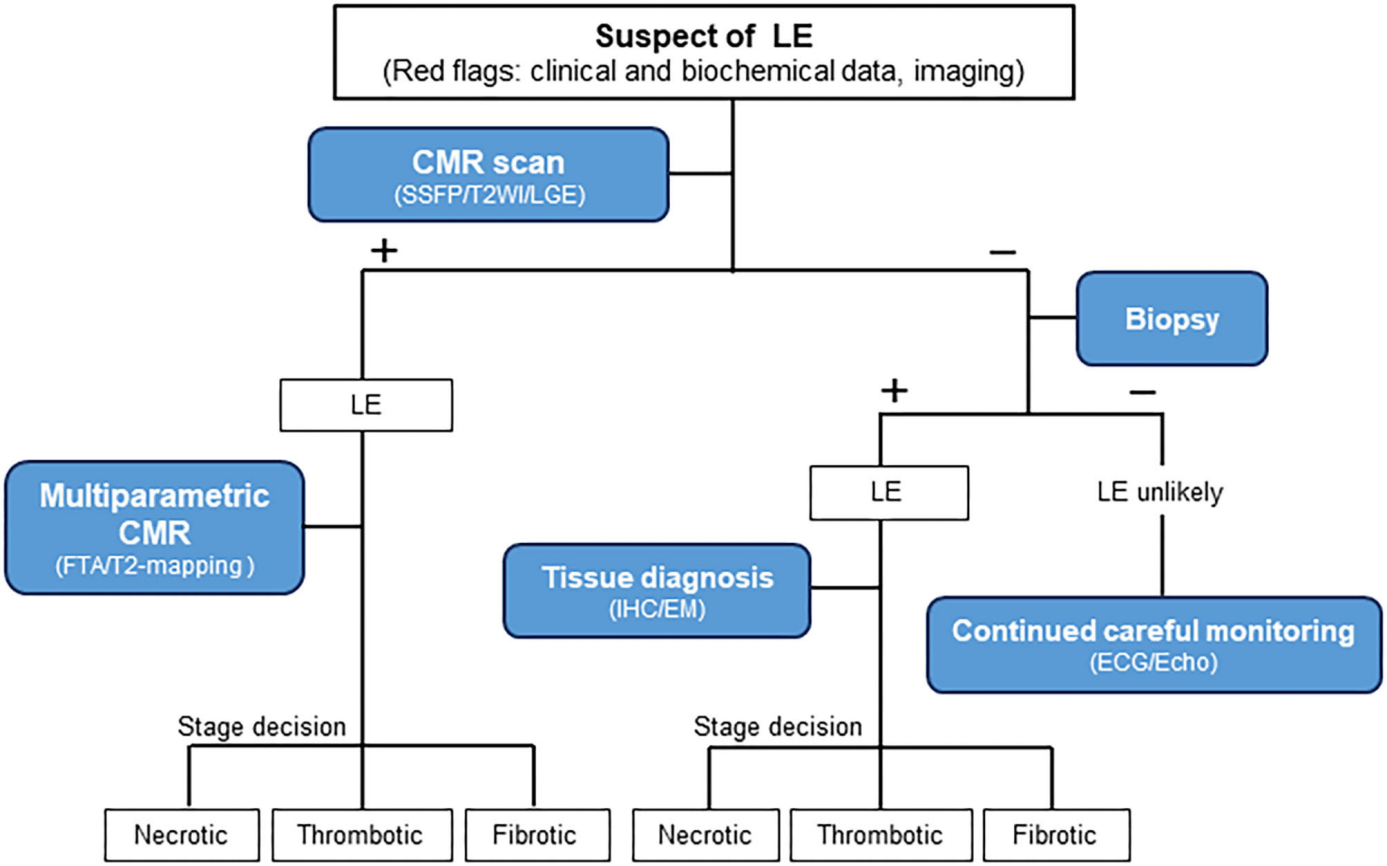
Diagnostic algorithm for patients with suspected Löffler's endocarditis. LE, Löffler's endocarditis; –, negative test; +, positive test; CMR, Cardiovascular magnetic resonance; SSFP, Steady-state free precession; T2WI, T2-weighted imaging; LGE, Late gadolinium enhancement; FTA, Feature-tracking based strain analysis; IHC, Immunohistochemistry; EM, Electron microscopy; ECG, Electrocardiogram; Echo, Echocardiography.

Thus, we propose a diagnostic algorithm for suspected LE using a comprehensive approach comprising clinical and biochemical data, multimodality imaging, and careful monitoring (Figures 6, 7). LE should be highly suspected if persistent hypereosinophilia in combination with one or more red flags is observed. Subsequent multiparametric CMR allows non-histological diagnosis, disease staging, and evaluation of treatment response reliably. If necessary, clinicians should not hesitate to perform an endomyocardial biopsy to obtain additional information. Continuous careful monitoring is essential even if a single test is negative for LE.

## Conclusion

We described a case of histologically proven late-stage LE presenting as a cardiac mass, which was successfully treated with the combination of surgical intervention and sequential medical treatment. Clinicians should be aware of pitfalls in multimodality imaging of thrombus, and should consider the possibility of this rare clinicopathological entity in the differential diagnosis of cardiac mass. A comprehensive approach comprising clinical and biochemical data, multimodality imaging, and careful monitoring can be helpful for detecting LE.

## Data Availability Statement

The original contributions generated for this study are included in the article/Supplementary Material, further inquiries can be directed to the corresponding author/s.

## Consent for Publication

The authors confirm that written consent for submission and publication of this case report, including the images and associated movie, has been obtained from the patient.

## Author Contributions

TK was responsible for the clinical design and conceptualization. TK, MM, TI, and ST were involved in the acquisition of the clinical data. TK, HY, MM, and JI analyzed and interpreted the data. TK and HY wrote the manuscript. All authors discussed, read, and approved the submission of this manuscript to the journal.

## Conflict of Interest

The authors declare that the research was conducted in the absence of any commercial or financial relationships that could be construed as a potential conflict of interest.

## Supplementary Material

Supplementary Video 1 Transthoracic echocardiography in the apical 5-chamber view shows a markedly enlarged cardiac mass originating from the mid-anteroseptal wall of the left ventricle, extending to the anterolateral papillary muscle.

Supplementary Video 2 Color Doppler echocardiography in apical 3-chamber view shows a heterogenous cardiac mass with entrapment of the anterolateral papillary muscle, causing moderate mitral regurgitation.

## Abbreviations

HES: Hypereosinophilic syndrome
LE: Löffler's endocarditis
HF: heart failure
MR: Mitral regurgitation
ECG: Electrocardiogram
ALPM: Anterolateral papillary muscle
CMR: Cardiac magnetic resonance
LGE: Late gadolinium enhancement
MVR: Mitral valve replacement
EMF: Endomyocardial fibrosis
BNP: Brain natriuretic peptide
CRP: C-reactive protein
cTnI: Cardiac troponin I.
